# Neural Tube Defects: From a Proteomic Standpoint

**DOI:** 10.3390/metabo5010164

**Published:** 2015-03-17

**Authors:** Tania M. Puvirajesinghe, Jean-Paul Borg

**Affiliations:** 1 CRCM, Cell Polarity, Cell signalling and Cancer, Equipe labellisée Ligue Contre le Cancer, Inserm, U1068, Marseille F-13009, France; E-Mail: tania.guenneau-puvirajesinghe@inserm.fr; 2 Institut Paoli-Calmettes, Marseille F-13009, France; 3 Aix-Marseille University, F-13284 Marseille, France; 4 The National Center for Scientific Research, CNRS, UMR7258, F-13009, France

**Keywords:** neural tube defects, proteomics, planar cell polarity

## Abstract

Neural tube defects (NTDs) are congenital birth defects classified according to their resulting morphological characteristics in newborn patients. Current diagnosis of NTDs relies largely on the structural evaluation of fetuses using ultrasound imaging, with biochemical characterization used as secondary screening tools. The multigene etiology of NTDs has been aided by genetic studies, which have discovered panels of genes mutated in these diseases that encode receptors and cytoplasmic signaling molecules with poorly defined functions. Animal models ranging from flies to mice have been used to determine the function of these genes and identify their associated molecular cascades. More emphasis is now being placed on the identification of biochemical markers from clinical samples and model systems based on mass spectrometry, which open novel avenues in the understanding of NTDs at protein, metabolic and molecular levels. This article reviews how the use of proteomics can push forward the identification of novel biomarkers and molecular networks implicated in NTDs, an indispensable step in the improvement of patient management.

## 1. Introduction—Inborn Errors of Development: Neural Tube Defects

Neural tube defects (NTDs) are congenital anomalies of the central nervous system and rank amongst the most common birth defects alongside congenital heart anomalies and genito-urinary defects [1]. NTDs define a group of severe congenital malformations of the central neural system resulting from failure of the neural tube to close during neurulation between 21 and 28 days after conception [2]. Prevalence statistics show 5 per 10,000 in the United States in 2001–2004 and 10 to 15 per 10,000 in Western Australia in 2001–2006 individuals were affected. In certain countries such as China, NTDs are more prevalent with studies showing 20 per 1000 in 2002–2004 [3]. NTDs have a multifactorial etiology, arising from multiple interacting genes and environmental factors [4]. Both genetic and non-genetic factors are involved in the etiology of NTDs, with up to 70% of the variance in NTD prevalence due to genetic factors [5].

The formation of the neural tube or neurulation, is the embryonic process that leads to the ultimate development of neural tube. This process can be broadly divided into 2 phases:

—The primary phase occurs in week 3–4 and involves the formation of the brain and neural tube from the caudal region to the upper sacral level [6].
—The secondary phase completes the distal sacral and coccygeal regions [6].

Primary neurulation is associated with “open” NTDs and result in conditions including anencephaly, myelomeningocele (open spina bifida) and craniorachischisis. Conditions of NTDs which are associated with skin covering lesion sites of the spinal cord structure such as asymptomatic spina bifida occulta and severe spinal cord tethering are classed as “closed” NTDs. These are traceable to disruption of secondary neurulation [7]. Primary neurulation in mammals has been defined by distinct anatomical closure sites, at the hindbrain/cervical spine (closure 1), forebrain/midbrain boundary (closure 2), and rostral end of the forebrain (closure 3) [8].

### 1.1. Clinical Features of NTDs

Historical recordings of neural tube defects date back to the ancient Egyptian periods, which could be explained by the unmistakable clinical features observed in fetuses and infants [9]. The clinical features of NTDs vary greatly. The two most frequent types of NTDs are anencephaly and myelomeningocele which appear in approximately 40 and 50 percent of NTDs detected in established pregnancies, respectively [10]. The clinical features of anencephaly are the lack of brain and cranial vault which are associated with fetal loss or stillbirth. Myelomeningocele is associated with an open spinal cord protected only with the meningeal sac (SB cystica) or completely exposed (known as spina bifida aperta). Myelomeningocele is usually associated with live births, with symptoms including hydrocephalus which can cause increased intracranial pressure inside the skull and progressive enlargement of the head and mental disability [10]. Craniorachischisis is the most severe disorder of primary neurulation, clinically characterized by the complete absence of skull and extensive defects in the vertebrae and skin. Most cases are associated with spontaneous abortion early in pregnancy [11]. NTDs are associated with very poor life expectancy statistics with 20 percent of individuals dying in utero (as stillbirths or therapeutic abortions). The remainder of individuals survive beyond the first week of life with approximately 10 percent of individuals dying within the first year. Those living beyond this period hold the promise of a life of poor health and repeated medical and surgical interventions and physiotherapy [7].

### 1.2. Standard Procedures for the Diagnosis NTDs

The standard operating procedure for the diagnosis of open and closed NTDs (OCNTDs) including anencephaly, encephalocele, and spina bifida is primary screening for detection of fetal structural abnormalities. Undertaken in the second trimester, anatomical (high-resolution) ultrasound is used to obtain a detailed image for fetal intracranial and spine assessment [12]. Ultrasound screening has now surpassed the original gold standard alpha fetoprotein (AFP) biochemical test because ultrasound screening is reported to have up to 97% sensitivity and 100% specificity in the diagnosis of NTDs in the hands of experienced ultrasonographers. The major pitfall of ultrasonography is the high false-negative rate and the technique is therefore potentially dangerous in the hands of less experienced ultrasonographers [13]. For this reason the dosage of AFP in maternal serum is used as a secondary screening tool to detect NTDs in certain clinical cases for instance when pregnant women have a pre-pregnancy body mass ≥ 35 kg/m^2^ or when geographical parameters may limit the use of good quality ultrasound screening at 18–22 weeks gestation [14].

Normal operating procedures for the management of NTDs begin with the initial diagnosis of a fetus with a NTD in the second trimester. Patients are referred to regional or tertiary centers for more detailed evaluation using more sophisticated ultrasound examination equipment. Prenatal magnetic resonance imaging can be proposed at this point if central nervous system examination is required. The use of invasive tests is then considered including amniotic fluid analysis following amniocentesis to analyze fetal karyotype, amniotic fluid AFP and amniotic fluid acetylcholinesterase. The utility of amniocentesis is carefully considered before its use due to the risk of spontaneous fetal loss, which occurs in 1 in every 200 procedures [15]. Pregnancy management given to families includes one of 3 options, consisting of:

—prenatal myelomeningocele repair and prognosis
—postnatal myelomeningocele surgical repair and prognosis
—pregnancy termination with autopsy [12].

Studies have shown that a combination of screening strategies using both ultrasound and biochemical characterization are important to achieve high detection rates [16]. For patients identified to have a higher risk of NTDs, obstetricians now use combination test strategies for screening referred to as “triple”, “quad” or “penta” screening tests depending on the number of analytes measured in maternal serum and produced by the fetus and the placenta. A risk estimate is then calculated using a mathematic model, which takes into consideration maternal demographic information (age, weight, gestational age, diabetes status and race). Different analytes measured include AFP, *human chorionic gonadotropin* (hCG) and unconjugated estriol (μ *E3*), which are measured using a variety of biochemical techniques including enzyme immunoassay and luminometric tests. Biochemical tests are carried out in the second trimester and so offer no advantage of early detection.

Financially, ultrasound detection and treatment management of NTDs pose a huge economic burden to healthcare systems [17]. Therefore there is renewed emphasis on the early detection of NTDs along with the development and use of less expensive tests for the prediction of disease severity. This would be beneficial for those associated with a high probability of NTD-affected pregnancy and would allow more time for informed decisions to be taken to ensure healthy outcomes for mother and fetus. Indeed the genetic disposition of NTDs has shown that there is an increased risk of NTDs after a miscarriage. Therefore there is common recognition, *acceptance* and *appreciation* for the need for earlier detection and ultimately the development of pharmacological treatments for *in utero* closure of NTDs [18].

### 1.3. Determining the Causative Factors or Contributing Factors of NTDs

#### 1.3.1. Importance of Vitamins and Vitamin-Related Genes

One of the first suspected causes of NTDs was vitamins. Vitamins including folate were initially shown to be important in NTDs with the initial observation that mothers pregnant with NTD fetuses had lower serum folate levels compared to controls patients [19] and epidemiological studies showing that folate deficiency along with vitamin B12 deficiency are risk factors for NTDs [20]. Studies show that folate supplementation can prevent NTDs in certain genetic mouse models including *Crooked tail*, *Splotch* and *Cited2* [21,22,23,24]. However, accumulated experimental evidence argues against a simple folate-deficiency model [7]. This is shown by the fact that maternal folate levels in affected pregnancies are within the “normal” range and evidence gathered from rat models which were unaffected by NTDs as a result of FA deficiency (reviewed in [25]).Therefore, sub-optimal folate status may pre-dispose to NTDs in combination with additional factors, either environmental or genetic.

#### 1.3.2. Importance of Methylation-Related Genes

One important genetic factor related to one-carbon metabolism of folate is methylenetetrahydrofolate reductase (MTHFR). MTHFR catalyzes the conversion of methylenetetrahydrofolate (CH_2_H_4_folate) to methyltetrahydrofolate (CH_3_H_4_folate). The CH_3_H_4_ folate is utilized as the methyl donor in the conversion of homocysteine to methionine catalyzed by the vitamin B12-dependent enzyme methionine synthase. A polymorphism mutation of C677T which replaces an Ala222 with Val in the human enzyme leads to a thermolability of MTHFR [26,27] and mild elevation of plasma homocysteine levels, a parameter associated to NTDs (reviewed in [28]). The presence of the polymorphism mutation of C677T variant in children with spina bifida or their mothers increases the risk of having an NTD or having a child with an NTD, respectively. A large-scale meta-analysis study including 2429 cases and 3570 controls suggests that maternal MTHFR C677T polymorphisms are a genetic risk factor for NTD [29,30,31].

However the prevalence of the MTHFR C677T polymorphism varies depending on geographical and racial/ethnic variation. In Europe alone, the prevalence of this polymorphism varies between 10–26 percent [32]. The high risk of false-positive detections and the risk of related anxiety that knowledge of being a carrier of the given mutation may bring to a patient, mean that screening for MTHFR C677T polymorphisms is not carried out.

#### 1.3.3. Importance of Metabolites

Elevated serum levels of the thiol-containing amino acid, homocysteine, are associated with NTDs (reviewed in [28]). Homocysteine binds to protein cysteine residues, which are required for diverse roles including protein folding and regulation of the quaternary structure through the formation of disulphide bonds. Homocysteine binding also regulates the catalytic domains of proteins which in turn regulate other cellular functions including cell signaling, transcription and protein trafficking [33].

### 1.4. Use of Animal Models for the Study of Different Types of NTDs

In the laboratory, the use of animal models has been instrumental in the identification of causative disease factors in NTDs. Mice studies have also been employed in order to elucidate the causative effects of NTDs in more complex vertebrate systems. More than 200 genetic models of NTDs have been described in mice, which include examples of the main open NTD phenotypes namely anencephaly, open spina bifida and craniorachischisis. These models have provided invaluable information concerning the molecular signaling pathways and cell biological processes in neurulation [34].

**Figure 1:**
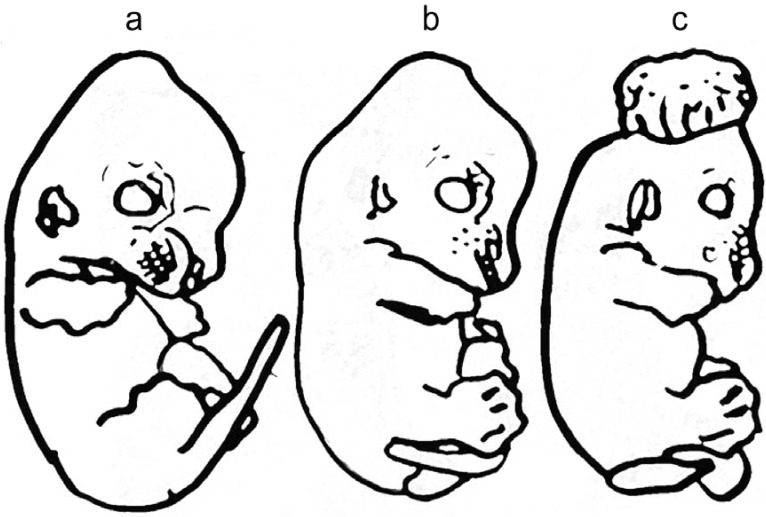
Representation of scanning electron micrographs of E13 curly tail mouse embryos suffering from neural tube defects (NTDs) (adapted from [35]): (**a**) embryo classified as unaffected phenotype; (**b**) embryo showing spina bifida and dorsally flexed tail; (**c**) embryo of severely affected phenotype showing exencephaly.

The most extensively studied mouse model is the curly tail (ct) model [35], initially described in 1954 by Hans Grunberg. Curly tail provides a useful mouse model for comparison with humans due to common features including partial penetrance with a major influence of genetic modifiers and environmental factors [35]. These mice have a high incidence of spina bifida and distinct morphological abnormalities including kinked and curled tails, which are attributed to homozygous loss of function of a recessive gene (ct) [36] and shown in Figure 1. The ct genetic defect has been mapped by linkage studies using microsatellite markers to chromosome 4 to the region containing grainyhead-like-3 (Grhl3) (reviewed in [37]). A mutation in the upstream enhancer element of Grhl3 has been shown to be responsible for the development of spina bifida in ct/ct embryos [38]. Grhl3 is a transcription factor essential for neural tube closure as Grhl3 knockout mice (Grhl3-/-) develop NTD with a 100% penetrance and mice with hypomorphic expression of Grhl3 do not survive [39,40,41]. Grhl3 belongs to the Grh/CP2 family of proteins which are involved in transcription regulation due to their multidomain protein structure which includes dedicated regions for DNA binding and protein dimerization. Grhl3 regulates expression of proteins involved in neural tube closure and is involved in other crucial biological processes such as epidermis development and wound healing [42].

### 1.5. Defects of Planar Cell Polarity Play a Causative Role in NTDs

NTDs are studied by analyzing convergent extension (CE) processes, which describe the collection of morphogenetic movements and cell behaviors that contribute to narrowing and elongation of the embryonic body plan [43]. The initiation process of neural tube closure has shown to be regulated by the embryonic CE process [44]. In vertebrates, CE is responsible for the polarization of entire fields of cells in a plane perpendicular to the apical-basal axis and planar cell polarity (PCP) is the key regulator of CE gastrulation movements [45]. A delicate balance of PCP levels is shown to be a crucial requirement with both gain- and loss-of function of PCP components resulting in the impairment of CE without affecting cell fate (reviewed in [46]). Mutations of mouse PCP genes cause a variety of NTDs including craniorachischisis, and sometimes spina bifida (SB) or exencephaly (EX); they also demonstrate the role of digenic combinations of PCP mutants in NTDs [47]. The most severe form of NTD, craniorachischisis, gives rise to an open neural tube from the midbrain to the tail and severe reduction in axial elongation [48]. Extensive studies have now characterized the causative effect of several PCP gene alterations in NTDs (Table 1) [49,50,51,52,53].

**Table 1:** Summary of mutations in planar cell polarity (PCP) genes and their phenotypes in mice.

PCP Genes	Phenotypes in Homozygous Mutant Mice
*Celsr*	Craniorachischisis
*Dvl1/2 or DVL2/3*	Craniorachischisis
*Fz3/6*	Craniorachischisis
*Pk1*	None
*Ptk7 (CCK-4)*	Craniorachischisis
*Scrib*	Craniorachischisis
*SEC24b*	Craniorachischisis
*Vangl1*	None
*Vangl2*	Craniorachischisis

Adapted from [10].

Mutations in PCP genes have been shown to be relevant in human NTDs through the use of mutation screening studies with case-control designs using ethnic-matched population controls. This demonstrates that mutations in genes VANGL1 (MIM# 610132) and *VANGL2* (MIM# 600533) play important roles in human NTDs [54,55,56]. Rare mutations of *VANGL2* have been shown to play critical roles in lethal human forms of NTDs [56]. Screens are now being established to identify non-synonymous mutations in *VANGL* genes that may play a role as risk factors in NTDs [57]. Missense mutations are also found in other genes capable of deregulating PCP including *DACT1* [58]. Mutations have shown to be a risk factor in human NTDs as missense mutations in DACT1 (MIM# 607861) have been functionally characterized to participate to PCP by regulating Dvl2 and JNK N-terminal kinase [59]. One study has shown that rare-mutations in PCP genes including *VANGL1*, *VANGL2*, *DVLs*, *CELSR*, *FZD6*, *PTK7* and SECb have been detected in 15 percent of tested human NTD cases and absent in 400 control cases, thus justifying the potential use of screening patients for PCP as risk factors [59].

Mouse models also provide information of different closure sites as studies in mice have identified three closure points involved in primary neurulation [60]. Mouse models from transcriptional factor Grlh3 and its other homologues Grlh2 are important in failure closure site 2 and 3. Embryos in which closure site 2 fails exhibit exencephaly involving the midbrain/hindbrain regions, whereas failure of closure site 3 leads to exencephaly involving the forebrain and extending to the mid/hindbrain, in association with a split-face malformation [61]. Closure site 1 is associated with PCP genes such as *VANGL2*. Hence closure site 1 is associated with signaling components which regulate PCP and not transcriptional activity.

When extrapolating biological data from animal models to understand human NTDs, one should carefully consider the type of model and study used. Indeed mouse and human embryos share similarities in certain closure sites including closure site 1, which occur in both species in the hindbrain/cervical boundary and closure spreads bidirectionally from this site [61]. Therefore future approaches could focus on genes implicated in mutations involved in closure site 1 in order to search for molecular biomarkers, which would need to be validated in human samples obtained by avoiding the destruction of healthy tissue (for example serum and urine). Nonetheless, differences occurring from the localization of closure sites 2 and 3 between humans and mice put into question conclusions gained from mouse models associated with closure 2 and 3 [60]. In addition, investigating human samples obtained from invasive procedures are associated with problems related to the small quantity of samples available from human embryos. This then poses problems for the application of proteomic and transcriptomic approaches to analyze human samples. In addition, inconsistences between animal and humans mean that research cannot be potentially translated into the clinic. One example is *MTHFR* knockout mouse embryos that do not develop NTDs but *MTHFR* variants remain a major risk factor in human NTDs [62].

### 1.6. Proteomics to Understand NTDs

Proteomics is a term coined in 1996 by Wilkins *et al.*, [63] and defines the large scale analysis of proteins in a cell, tissue or whole organism at a given time under defined conditions. Proteomics techniques have been gradually developing over the past 35–40 years and relates back to various research fields including that of protein separation, mass spectrometry, genome sequencing/annotation, and protein search algorithms. The advancement of proteomics techniques has gone hand-in-hand with technological advancements in mass spectrometers and associated analysis software [64]. Proteomics techniques can be broadly separated into gel-based and gel-free methods (which include chemical and metabolic labelling). The majority of studies employing MS techniques to study NTDs have used gel-based methods such as 2-D electrophoresis. In this case proteins are separated on the basis of their isoelectric point as well as its molecular weight. 2-D electrophoresis proteomic analysis techniques have been used to compare genetic strains affected by NTDs in order to discover biomarkers and causative factors of NTDs.

#### 1.6.1. Identifying Putative NTD Genetic Modifier Genes Using Proteomic Studies

The genetic component for the predisposition to NTDs is multifactorial with certain mouse models showing that frequency of NTDs is markedly affected by the backcross to different strains [65]. Proteomics studies have been useful in the identification of putative modifier genes. Studies have been carried out using the whole embryo samples as well as sub-specific regions of the neural tube (active neural tube) of embryonic samples collected from ct/ct homozygous mice and wild-type mice. Samples taken at the embryonic stage day 10.5 during the closure of the neural tube and separated using 2-D electrophoresis showed that a differentially migrated spot pattern was noted for ct/ct and +^ct^/+^ct^ mice. Following liquid chromatography tandem mass spectrometry (LC/MS), these changes were attributed to differences in lamin B1. Previous experiments analyzing mRNA levels in RT-PCR experiments and western blot analysis of whole proteins showed no difference in lamin B1 levels in wild-type and homozygous mutant mice. Isoelectric focusing was the only technique able to discriminate between variations in lamin B1 from ct/ct and +^ct^/+^ct^. This led to consequent sequencing of lamin B1, which identified a three base-pair GAG deletion in exon 10 [66] that results in eight Glu residues in the tail domain of the wild-type protein found at amino acids 553–560 (denoted as Lmnb^8E^) as opposed to nine Glu (residues 553–561) in the +^ct^/+^ct^. The negative charge of the Glu residue in turn explained the differential migrational status for lamin B1 in ct/ct homozygous mice [66]. Embryos expressing lamin B1 manifested defects in proliferation localized in the hindgut epithelium and showed diminished S-phase progression [66]. This study serves as an example of the proteomic identification of crucial modifier genes affecting NTDs.

#### 1.6.2. Comparative Proteomic Analyses in Rat/Animal Spina Bifida Model

Finer analysis of subsections of the spinal cord has also been carried out in rat models [67]. Neurological dysfunction can result directly from the primary defect in the neurulation process or from secondary injuries to the intrauterine environment such as exposure of the uncovered neural tissue at the site of the spinal defect to amniotic fluid, which is believed to be toxic [68,69]. Analysis of defective regions of the spinal cord can provide useful information on the status of proteins at different gestational states or conditions. In a proteomics study carried out in a chemically-induced (all-trans retinoic acids (ATRA)) rat spina bifida model, spinal cords of rat fetuses were analyzed in comparison to untreated rats [67]. Proteins were identified from this differential screen of malformed fetal spinal cords using 2-D electrophoresis and Matrix Assisted Laser Desorption Ionization Time-of-Flight mass spectrometry (MALDI-TOF MS) followed by validation by immunoblot analysis and quantitative real-time PCR. This study identified alterations in collapsin response mediator protein 4 (Crpm-4), heat shock protein-70 (Hsp-70) and calponin-3, a set of proteins implicated in signal transduction, transcriptional regulation, apoptosis and protein synthesis and folding. This type of strategy could lead to new opportunities for diagnosis and treatment of spina bifida in the future [67].

#### 1.6.3. Analysis of Metabolic Factors from Human Biological Fluids

Continued interest in folate and homocysteine-mediated one carbon metabolism and uncovering the molecular mechanism of disease have provided the rationale for using LC/MS techniques to analyze metabolites in maternal serum from NTD-affected pregnancies compared to control subjects. Metabolic profiling allowed the simultaneous analysis of 10 relevant components relating to folate vitamins. Significantly lower serum concentrations of different forms of folate such as 5-formyltetrahydrofolic acid (5-FoTHF) and higher levels of S-adenosylhomocysteine (SAH) can thus be used as potential risk factors for the diagnosis of NTDs [70].

Meta-analysis of the effectiveness of maternal serum AFP is reported to have the ability to detect 75 percent of NTDs during the second trimester of gestation. Though not useful as an earlier detection marker, this offers a cheaper, non-invasive and safe procedure to mother and fetus diagnostic test to supplement ultrasound imaging [71].

The multifactorial nature of NTDs has been analyzed using different biological fluids using Surface Enhanced Desorption Ionization-Time of Flight (SELDI-TOF MS). Fluids obtained using non-invasive procedures (serum or urine) as well as invasive procedures (amniotic fluid) have shown that NTDs-related pregnancies can be differentially identified using specific proteomic signatures [72]. The disadvantages of SELDI-TOF MS are that it can provide resolution of only small MW peptides and proteins (2–20 kD). However the SELDI-TOF MS technique is easily applicable to clinical trials.

Better understanding of folate metabolism has been the rationale for metabolic profiling studies analyzing the serum of NTD-affected pregnancies. Characterizing serum metabolic signatures have shown important differences in pyruvic acid and lactate mitochondria respiration as well as neurotransmitters [73]. Advantages include insights into the underlying metabolic mechanisms of NTDs with the potential application in the first trimester of gestation. Similar studies were carried out with placenta samples. Advantages are indeed the deeper understanding of different subtypes of NTDs which can each be discriminated by differential global metabolic profiles for one-carbon metabolism. A number of different metabolites were found in each NTD subtype and these metabolites are important in carbohydrate, amino acid, lipid and nucleic acid metabolism [74]. Translating the use of studying placenta samples is associated with drawbacks including the risk associated with invasive sampling of placenta and the difficulties in handling large numbers and small quantities of these samples in a clinical setting. Therefore, placenta analysis may be more important in a fundamental research setting in order to increase understanding of placental transport and the metabolic mechanisms underlying NTDs.

#### 1.6.4. Analysis of Amniotic Fluids

The biochemical composition of amniotic fluid varies during the different stages of pregnancy and can be used to monitor pathological and physiological changes of the mother and the fetus [75,76,77]. Biochemical analysis of amniotic fluid has been investigated using 2-D electrophoresis and MALDI-TOF MS in order to associate biochemical characteristics to pathological states, and establish disease-specific biomarkers. Due to the close similarity of the embryonic processes of neurulation in humans and rodents, animal models have been employed with the induction of spina bifida achieved by injection with ATRA [78]. Fetuses analyzed using MS techniques established up- or down-regulation of different proteins, for example up-regulation of AFP. In addition, MS methods were able to identify molecular variants of AFP and their prevalence. The study described 11 alpha-1 fetoprotein (AFP) fragments that were downregulated and 35 AFP fragments that were upregulated in AFSs from embryos with spina bifida aperta. MALDI-TOF MS analysis showed that from the downregulated AFP fragments, 72.7% (8/11) were confined to the AFP N-terminus (amino acids (aas) 25–440) and 77.1% (27/35) of upregulated AFP fragments were confined to the AFP C-terminus (aas 340–596) [78]. In humans, AFP variants have been related in publications dating back to 1970s [79]. Variants were attributed to carbohydrate moieties [80] and mRNA splicing [81]. Therefore the finer analysis of AFP variants may have important applications in diagnosis and for the design of therapeutic AFP-derived peptides [78].

Amniotic fluid has been used to investigate both amniotic fluid stem cells (AFSCs) and secreted growth factors to characterize the composition of amniotic fluid from different gestational stages. AFSCs isolated from primary cultures of amniotic fluids and grown into three-dimensional neurospheres showed that AFSCs deriving from amniotic fluid from myelomeningocele-affected human pregnancies were associated with higher levels of histone methylation, including methylation on H3K4me and H3K27me3 [82]. Amniotic fluid from myelomeningocele-affected human pregnancies showed higher levels of developmental proteins including BMP4 and Shh [82]. Changes in BMP4 and Shh were further confirmed in maternal serum [82]. Translation of this research could be useful in using developmental proteins such as BMP4 and Shh as biomarkers for early detection of NTDs from non-invasive samples such as maternal serum.

#### 1.6.5. Detection of Human Polymorphisms in Clinical Samples Using Proteomics Studies

The importance of aetiological factors of genes in one-carbon metabolism has already been described (Section 1). Proteomic studies using individuals with risk factors such as the thermolabile mutant forms of MTHFR compared to individuals with the wild-type genotype have been used to detect further potentially important polymorphisms in genes such as vitamin D related proteins. Analysis of human plasma samples has been carried out using 2-D electrophoresis and MS/MS [83]. There are 3 major allelic variants of human vitamin D binding protein also known as group component Gc globin (Gc) [84,85]. Proteomic analysis showed that MS/MS analysis was capable of discriminating between the 3 allelic variants of vitamin binding protein in the population [83]. MS/MS allowed the detection of the presence of the allelic variant Gc2 (group component Gc2) in mutated individuals, which corresponds to a lysine residue at position 420 compared to the common threonine residue (allelic variant Gc1F). Therefore MS/MS peptide sequencing can be used as a tool for the detection of polymorphisms in different clinical groups. Individuals with the *Gc2* allelic variant have a low vitamin D binding isoform of DBP resulting in functional consequences. Hence these conclusions expand the list of allelic variants associated to at-risk individuals, which is important in the potential screening of at-risk individuals using noninvasive biological fluid assays. However, further studies using a large number of samples would be necessary to validate the potential of screening individuals for Gc variants to identify NTDs.

#### 1.6.6. Using Affinity Based Strategies to Analyze Protein Networks

Protein signaling pathways involved in neural defects have been elucidated by affinity purification and mass spectrometry techniques. This has been shown with diseases such as nephronophthisis (NPHP), Joubert (JBTS) and Meckel-Gruber (MKS) syndromes [86] which present neural tube malformations as well as ciliopathies such as Bardet-Biedl syndrome [87]. To better identify physical interactions, an affinity purification strategy (G-LAP-Flp) has been employed with expression of inducible LAP-tag proteins (EGFP-TEV-S-peptide) fused to the N-terminus of individual disease proteins including NPHP1, NPHP2/Inversin, MKS1 amongst others proteins from different cell lines [88]. Tandem affinity purification and high-confidence proteomics techniques have been used to define protein partners. Resulting cluster protein signaling networks categorize proteins according to their biochemical and functional and into different groups (according to their subcellular localization in the cilia. This type of analysis has helped in the discovery of new disease causing genes, which has been important in suggesting potential therapeutic targets.

**Figure 2:**
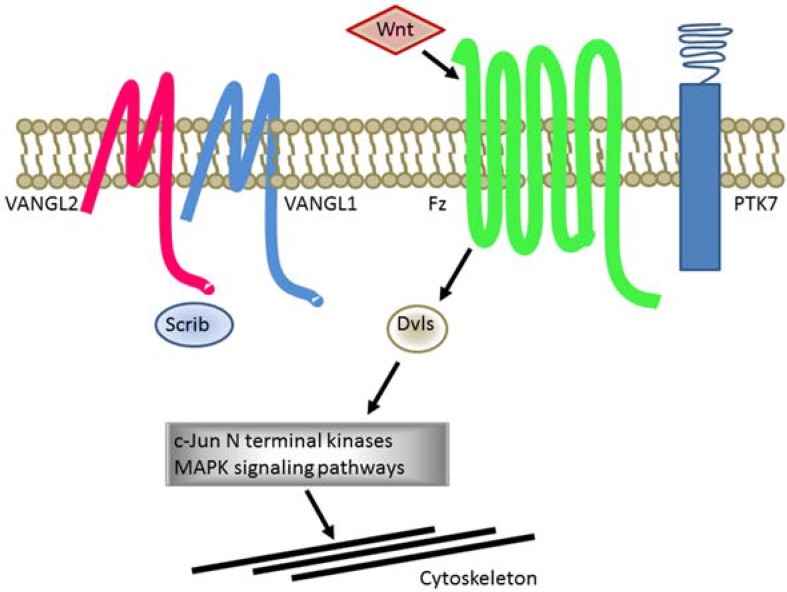
PCP proteins have causative effects in NTDs. *Schematic* illustration of PCP proteins VANGL2 and paralog VANGL1 alongside other PCP proteins including Frizzled (Fz), PTK7, Scrib and Disheveled (Dvls). Downstream signaling of PCP implicates c-Jun signaling cascades results in cytoskeleton rearrangements.

PCP is regulated by receptors (VANGL1, VANGL2, Frizzled, PTK7 among others) associated to downstream signaling proteins such as Disheveled, activation of JNK and cytoskeletal reorganization (Figure 2) [89]. Certain PCP protein components contain well-characterized protein interaction domains including PSD95-Discs Large-Zonula Occludens (PDZ) domains, which bind the C-terminal of proteins and induce clustering and signaling [90]. Different approaches using yeast two-hybrid techniques have identified several PDZ-containing proteins able to interact with VANGL2. Interactions were further validated by biochemical assays including peptide pulldown and co-immunoprecipitation experiments [91]. This has resulted in the identification of known PDZ proteins known to cause PCP including Scrib [92] and proteins not originally considered to play a role in PCP such as SNX27. This PDZ protein was subsequently described to have a morphogenetic function in the early Xenopus embryo.

The study of proteins involved in PCP has relied on the use of ectopic expression of proteins and Tag-based purification methods (including GFP, HA, Tap-tag techniques) [91,93]. Endogenous biochemical purification has been hampered by technical difficulties in distinguishing between members of the *VANGL* family of proteins, VANGL1 and VANGL2, due to their extreme amino identity and similarity. With the advent of the generation of monoclonal antibodies, immunopurification using antibodies specifically recognizing VANGL2 has been achieved. This provided the first detection and comprehensive biochemical characterization of the endogenous forms of these PCP proteins, demonstrating the ability of VANGL2 and VANGL1 to physically associate through their transmembrane domains to form a protein complex [94]. Through the development of purification tools and the technological improvement in the sensitivity of MS detection, affinity-based techniques can now be applied to biologically important samples. Limitations in affinity-based strategies remain incompatible with humans due to the lack of sample availability for analysis. However their future application to different mouse models may provide information on signaling and functional protein cascades which could indirectly lead to their potential use as biomarkers. The acquisition of different biological samples from different conditions and different cellular locations is an important aspect which should be highly investigated and controlled. This is potentially very important in conserving the dynamic nature of protein-protein complexes.

#### 1.6.7. Experimental and Computational Strategies Used for the Analysis of Diseases

Research into cardiovascular diseases has provided some interesting insights into MS experimental strategies used to study disease characteristics of *atherosclerotic lesions*. By analyzing the “secretome” of these lesions, it was noted that Hsp27 expression was correlated to the complexity of the artherosclerotic plaque [95]. Strategies combining direct tissue proteomics with laser capture microdissection have been used to provide large scale analysis of human coronary artherosclerotic tissues. This strategy was able to achieve identification of proteins from different tissue preparations of coronary arteries, including paraffin-embedded, paraformaldehyde and frozen sections [96]. Proteomics studies can therefore help to understand complex human diseases. Such procedures may theoretically be applied to the study of NTDs.

Elevated levels of the thiol-containing amino acid, homocysteine, is associated with NTDs. Computation models have been utilized to predict the reactivity of cysteine residues in proteins according to parameters such as accessibility of cysteine in a protein, pKa of free cysteines, and the dihedral strain energy (DSE) of a cysteine disulfide [97]. Using these predictive physiochemical parameters, proteins previously shown to cause NTDs were identified by such predictive models, including Notch [98] and Apolipoprotein B [99]. Though an extended list of proteins is not yet available, computational models can now provide physiochemical guidelines important in building larger predictive databases, which could help predict yet undescribed proteins important in NTDs caused by homocysteine induced pathogenesis. Ascribed models can also be employed to analyze pathways and networks potentially deregulated by homocysteine reactivity as well as analyze differential disease susceptibility to similar levels of homocysteine in different populations by integrating cysteine-specific polymorphisms data [97].

### 1.7. Discussion and Conclusions

Neural tube defects are common congenital anomalies associated with various detrimental clinical features. Genetic studies using different animal models have been central in understanding the causative factors of the multifactorial etiology of NTDs. Clinical diagnosis of NTDs relies upon high-resolution ultrasound for detection of fetal abnormalities in the second trimester of pregnancy. However alternative methods that rely on biomarkers would be useful in reducing the costs of diagnostic techniques, providing earlier detection of NTDs and advance the development of in utero pharmacological treatments. Somewhat surprisingly, given that AFP diagnosis began in 1976 is the fact that only a small number of publications document the use of proteomics techniques for the detection of NTDs. However advances have been made in the differential analysis of proteins, discrimination of polymorphisms in populations, and identification of different protein factors and analysis of protein networks for use in neural tube defects. In the future, application of bioinformatics tools may be applied to understand the molecular mechanisms of NTDs, which may also help understand their relevance in regulating other cellular processes including cancer [93,100,101,102]. Computational models are also capable of formulating the generalized effect of different protein functions. These models have been developed based on structural and physiochemical properties of protein cysteine residues in order to study the number of direct homocysteine binding proteins [97]. In this way, the complex effect caused by a single metabolite can be monitored to understand cellular signaling pathways, which have the potential to predict metabolite-induced pathogenesis.
